# Self-Reported Concussions from Playing a Sport or Being Physically Active Among High School Students — United States, 2017

**DOI:** 10.15585/mmwr.mm6724a3

**Published:** 2018-06-22

**Authors:** Lara DePadilla, Gabrielle F. Miller, Sherry Everett Jones, Alexis B. Peterson, Matthew J. Breiding

**Affiliations:** ^1^Division of Unintentional Injury Prevention, National Center for Injury Prevention and Control, CDC; ^2^Division of Analysis, Research, and Practice Integration, National Center for Injury Prevention and Control, CDC; ^3^Division of Adolescent and School Health, National Center for HIV/AIDS, Viral Hepatitis, STD, and TB Prevention, CDC.

Increased susceptibility to concussions and longer recovery times among high school athletes compared with older athletes (*1*) make concussions among youths playing a sport or being physically active an area of concern. Short-term and long-term sequelae of concussions can include cognitive, affective, and behavioral changes (*1*). Surveillance methods used to monitor concussions among youths likely underestimate the prevalence. Estimates assessed from emergency departments miss concussions treated outside hospitals, those generated using high school athletic trainer reports miss concussions sustained outside of school-based sports (*2*), and both sources miss medically untreated concussions. To estimate the prevalence of concussions among U.S. high school students related to playing a sport or being physically active, CDC analyzed data from the 2017 national Youth Risk Behavior Survey (YRBS). Overall, 15.1% of students (approximately 2.5 million*) reported having at least one of these concussions during the 12 months before the survey, and 6.0% reported two or more concussions. Concussion prevalence was significantly higher among male students than among female students and among students who played on a sports team than among students who did not. Among all sex, grade, and racial/ethnic subgroups, the odds of reporting a concussion increased significantly with the number of sports teams on which students played. These findings underscore the need to 1) foster a culture of safety in which concussion prevention and management is explicitly addressed; 2) expand efforts to educate students, parents, coaches, and health care providers regarding the risk for concussion; and 3) identify programs, policies, and practices that prevent concussions.

YRBS is a biennial, cross-sectional, school-based survey that uses a three-stage cluster sampling design to produce nationally representative samples of public and private school students in grades 9–12 (*3*). In accordance with local parent permission procedures, students voluntarily completed an anonymous, self-administered questionnaire during one class period and recorded their responses on a computer-scannable answer sheet. An institutional review board at CDC approved the protocol for the national YRBS. In 2017, the school response rate was 75%, the student response rate was 81%, the overall response rate was 60%,^†^ and the sample size was 14,765.

In 2017, CDC included a question about concussions on the national YRBS questionnaire for the first time. Following a definition of concussion (“when a blow or a jolt to the head causes problems such as headaches, dizziness, being dazed or confused, difficulty remembering or concentrating, vomiting, blurred vision, or being knocked out”), students were asked, “During the past 12 months, how many times did you have a concussion from playing a sport or being physically active?” Response options were: “0 times, 1 time, 2 times, 3 times, and 4 or more times.” Sports team participation was assessed with the question, “During the past 12 months, on how many sports teams did you play? (Count any teams run by your school or community groups.)” Response options were “0 teams, 1 team, 2 teams, and 3 or more teams.” Prevalence estimates were computed overall and by sex (female or male), grade (9, 10, 11, or 12), the number of sports teams on which students played (0, 1, 2, or ≥3), and race/ethnicity (non-Hispanic white [white], non-Hispanic black [black], or Hispanic). The number of students in other racial/ethnic subgroups was too small for meaningful analysis.

Sampling weights were applied to each student record to adjust for nonresponse and the oversampling of black and Hispanic students. For all analyses, statistical software was used that took into account the complex sampling design and sampling weights. Chi-square tests were used to identify associations between student characteristics and having had 0, 1, 2, 3, or ≥4 concussions. T-tests were used for pairwise comparisons when a chi-square test result was significant. Among students who played on at least one sports team, the association between the number of teams on which students played and concussion was evaluated for each demographic subgroup using unadjusted logistic regression models. First, number of sports teams was treated as a categorical variable and then, to test for a linear association, as a continuous variable.

Overall, 9.1% of high school students reported one concussion, 3.0% reported two, 1.0% reported three, and 2.0% reported four or more concussions related to sports or physical activity during the 12 months before the survey (Table 1). Thus, 15.1% of students reported having at least one concussion, and 6.0% reported having two or more. Male students were more likely to report one, two, and four or more concussions than were female students. Students in grades 9, 10, and 11 were more likely to report a single concussion than were students in grade 12, and students in grade 9 were more likely to report a single concussion than were students in grade 10. Black and Hispanic students were more likely to report four or more concussions than were white students. Students who played on at least one sports team were more likely to report one, two, three, and four or more concussions than were those who did not play on any teams.

**TABLE 1:** Percentage of high school students who reported having a concussion from playing a sport or being physically active,* by number of concussions^†^ and selected characteristics — Youth Risk Behavior Survey, United States, 2017^§^

Characteristic	No. of concussions reported				
	0	1	2	3	≥4
	% (95% CI)	% (95% CI)	% (95% CI)	% (95% CI)	% (95% CI)
**Total**	**84.9 (83.3–86.4)**	**9.1 (8.2–10.2)**	**3.0 (2.5–3.5)**	**1.0 (0.8–1.3)**	**2.0 (1.6–2.4)**
**Sex**					
Female	87.0 (85.3–88.6)	8.0 (6.8–9.5)	2.6 (2.1–3.2)	0.8 (0.6–1.1)	1.5 (1.2–1.9)
Male	82.9 (81.1–84.4)	10.2 (9.2–11.3)	3.3 (2.6–4.1)	1.2 (0.9–1.6)	2.4 (1.9–3.1)
p-value	0.000	0.002	0.044	0.054	0.006
**Grade**					
9	83.0 (80.8–84.9)	11.3 (9.7–13.2)	3.0 (2.3–4.0)	0.9 (0.6–1.3)	1.8 (1.2–2.5)
10	84.8 (82.6–86.8)	8.5 (7.2–10.1)	3.2 (2.4–4.3)	1.4 (1.1–1.9)	2.0 (1.4–2.8)
11	84.7 (82.4–86.7)	9.6 (8.1–11.3)	3.0 (2.2–4.2)	0.9 (0.6–1.5)	1.8 (1.3–2.6)
12	87.8 (85.6–89.7)	6.8 (5.5–8.5)	2.5 (1.9–3.2)	0.7 (0.5–1.2)	2.1 (1.5–3.0)
p-value	0.001^¶^	0.001**	0.529	0.103	0.867
**Race/Ethnicity**					
White, non-Hispanic	85.4 (83.2–87.4)	9.7 (8.5–11.2)	2.6 (2.0–3.5)	0.9 (0.7–1.3)	1.3 (0.9–1.7)
Black, non-Hispanic	83.0 (80.7–85.1)	8.2 (6.7–10.0)	3.3 (2.6–4.2)	1.7 (1.1–2.8)	3.7 (2.1–6.3)
Hispanic	85.1 (83.2–86.8)	8.2 (7.3–9.3)	3.4 (2.6–4.6)	0.9 (0.6–1.3)	2.4 (1.7–3.4)
p-value	0.183	0.060	0.347	0.210	0.014^††^
**Played on at least one sports team** ^§§^					
Yes	78.6 (76.3– 80.7)	13.1 (11.7–14.6)	4.2 (3.5–5.1)	1.5 (1.2–2.0)	2.6 (2.0–3.2)
No	92.4 (91.2–93.5)	4.5 (3.7–5.4)	1.4 (1.0–1.9)	0.4 (0.3–0.6)	1.3 (1.0–1.7)
p-value	0.000	0.000	0.000	0.000	0.001

**Abbreviation:** CI = confidence interval.

* During the 12 months before the survey.

^†^15.1% of high school students reported at least one concussion.

^§^ Weighted percentages are presented. Weighted percentages might not add to 100 because of rounding.

^¶ ^Prevalence among 12th grade students was significantly higher than among 9th, 10th, and 11th grade students.

** Prevalence among 12th grade students was significantly lower than among 9th, 10th, and 11th grade students; prevalence among 9th grade students was significantly higher than among 10th grade students.

^††^ Prevalence among non-Hispanic white students was significantly lower than among non-Hispanic black and Hispanic students.

^§§^ Run by their school or community groups during the 12 months before the survey.

Among students who played on one, two, and three or more sports teams, the prevalence of reporting having had at least one concussion was 16.7%, 22.9%, and 30.3%, respectively (Table 2). Among students who played on at least one sports team, for all demographic subgroups, the odds of reporting a concussion increased with an increasing number of teams on which students played (Table 3).

**TABLE 2:** Percentage of high school students who reported having a concussion from playing a sport or being physically active,* by number of sports teams on which students played^†^ and selected characteristics — Youth Risk Behavior Survey, United States, 2017^§^

Characteristic	No. of sports teams on which students played			
	0	1	2	≥3
	% (95% CI)	% (95% CI)	% (95% CI)	% (95% CI)
**Total**	**7.6 (6.5–8.8)**	**16.7 (14.8–18.9)**	**22.9 (19.7–26.4)**	**30.3 (26.6–34.1)**
**Sex**				
Female	6.9 (5.5–8.7)	15.2 (13.3–17.4)	21.1 (18.1–24.5)	29.1 (23.7–35.2)
Male	8.3 (6.8–10.1)	18.3 (15.7–21.2)	23.9 (20.0–28.4)	30.9 (26.4–35.9)
**Grade**				
9	9.6 (7.5–12.4)	18.2 (14.5–22.7)	23.0 (18.5–28.3)	28.4 (23.3–34.1)
10	8.6 (6.9–10.7)	17.4 (13.8–21.6)	21.1 (16.4–26.9)	29.3 (23.0–36.4)
11	6.3 (4.6–8.7)	16.6 (12.1–22.3)	25.1 (20.0–31.0)	34.9 (29.1–41.2)
12	5.6 (4.2–7.3)	14.4 (11.7–17.5)	21.1 (16.7–26.4)	28.4 (21.2–36.9)
**Race/Ethnicity**				
White, non-Hispanic	6.6 (5.1–8.4)	15.7 (13.4–18.3)	22.9 (18.7–27.8)	30.8 (25.7–36.4)
Black, non-Hispanic	8.2 (6.4–10.5)	18.2 (14.5–22.5)	26.1 (20.1–33.2)	30.4 (23.0–38.9)
Hispanic	9.4 (7.6–11.7)	17.4 (13.8–21.8)	21.6 (17.9–25.7)	26.5 (21.2–32.5)

**Abbreviation:** CI = confidence interval.

* One or more times during the 12 months before the survey.

^†^ Run by their school or community groups during the 12 months before the survey.

^§^ Weighted percentages are presented. Weighted percentages might not add to 100 because of rounding.

**TABLE 3:** Association between the number of sports teams on which high school students played* and having reported a concussion from playing a sport or being physically active,^†,^^§^ by selected characteristics — Youth Risk Behavior Survey, United States, 2017

Characteristic	No. of sports teams on which students played			Linear trend^¶^ p-value
	1	2	≥3	
	OR (95% CI)	OR (95% CI)	OR (95% CI)	
**Total**	**Referent**	**1.5 (1.2–1.8)**	**2.2 (1.8–2.6)**	**<0.001**
**Sex**				
Female	Referent	1.5 (1.2–1.9)	2.3 (1.7–3.0)	<0.001
Male	Referent	1.4 (1.1–1.8)	2.0 (1.5–2.6)	<0.001
**Grade**				
9	Referent	1.3 (0.9–1.9)	1.8 (1.3–2.5)	0.001
10	Referent	1.3 (0.9–1.9)	2.0 (1.2–3.1)	0.007
11	Referent	1.7 (1.2–2.5)	2.7 (1.7–4.4)	<0.001
12	Referent	1.6 (1.1,2.3)	2.4 (1.6–3.6)	<0.001
**Race/Ethnicity**				
White, non-Hispanic	Referent	1.6 (1.2–2.1)	2.4 (1.8–3.1)	<0.001
Black, non-Hispanic	Referent	1.6 (1.1–2.4)	2.0 (1.3–2.9)	<0.001
Hispanic	Referent	1.3 (1.0–1.7)	1.7 (1.0–2.8)	0.027

**Abbreviations:** CI = confidence interval; OR = odds ratio.

* Run by their school or community groups during the 12 months before the survey.

^†^ Among students who played on at least one sports team during the 12 months before the survey.

^§^ One or more times during the 12 months before the survey.

^¶^ Logistic regression models tested a linear association between the number of sports teams on which students played and the odds of reporting having a concussion from playing a sport or being physically active.

## Discussion

In 2017, an estimated 2.5 million high school students reported having at least one concussion related to sports or physical activity during the year preceding the YRBS, and an estimated 1.0 million students reported having two or more concussions during the same time frame. The findings suggest that students who played on a sports team had a significantly higher risk for one or more concussions than did students who did not play on a team. Furthermore, concussions were significantly more common among students who played on two and three or more sports teams than among those who played on one team. The prevalence of having one or more concussions (15.1%) is comparable to the findings of an analysis of 2013 YRBS data from three states that added different questions to their survey to assess sports-related concussions among high school athletes. In that study, the prevalence ranged from 17.6% to 20.1% (*4*). The prevalence of concussions in the current study is higher than estimates based on emergency department data (e.g., 622.5 visits per 100,000 population aged 10–14 years) (*5*) and athletic trainer reports (e.g., 1.8 per 100 high school and college athletes for an average season) (*6*). Emergency department data miss concussions treated elsewhere, and athletic trainer reports miss concussions sustained outside of school-based sports; both sources miss medically untreated concussions (*2*).

Although increased awareness and recognition might result in higher rates of reported concussions (*1*), underreporting of concussions among athletes remains an important issue. A study of high school athletes found that among athletes with concussions, 40% reported that their coach was unaware of their symptoms (*7*). Students might not always recognize or remember that they have experienced a concussion, or they might not want to report having experienced a concussion. In this study, the opportunity to anonymously self-report a concussion, without negative consequences, such as a loss of playing time, might have aided in including concussions missed by other data sources. However, this study might overestimate the prevalence of concussions related to sports or physical activity if students reported concussions occurring before the 12-month reference period (*8*) or mistakenly thought that they had a concussion because some symptoms of a concussion, such as a headache, also occur in the absence of a concussion (*9*).

The findings in this report are subject to at least four limitations. First, these concussions, self-reported by students, were not validated (e.g., through medical record review). Second, these data apply only to high school students who attend school and are not representative of all youths in this age group or in other age groups. Nationwide, in 2013, approximately 5% of persons aged 16–17 years were not enrolled in high school and lacked a high school credential.^§^ Third, continuing to play sports or be physically active with a concussion that is symptomatic increases the risk for a subsequent, more serious concussion (*10*). YRBS data found that 6% of students reported two or more concussions, but YRBS data do not allow for determining the proportion of concussions that might have been related to a previous concussion that had not fully healed. Finally, it is not known what proportion of concussions occurred during team sports participation versus other types of physical activity.

A 2014 National Academy of Sciences report concluded that, in light of the limitations of existing surveillance systems, more comprehensive estimates of youth sports concussions are needed (*1*). This study, and state-level YRBS data (*4*), support this conclusion by suggesting that many concussions among youths are not being counted and might also indicate that similar comprehensive estimates below the high school level are needed. To that end, CDC is working toward developing a National Concussion Surveillance System to determine the incidence and identify the circumstances of concussions, and all traumatic brain injuries, across the lifespan (*2*).

The findings in this report support the need to continue education efforts addressing concussion risk associated with sports and physical activity, and indicate a need for messaging targeted toward students who play on multiple sports teams. Additionally, black and Hispanic students were more likely to report four or more concussions than were white students; targeted messaging might be needed to educate these groups in particular about the risks associated with sustaining multiple concussions. In addition, coaches and parents can encourage athletes to follow the rules of play for their sport with an emphasis on player safety, which might reduce the incidence and severity of concussions (*1*,*10*). It is important that any athlete with a suspected concussion be removed from practice and competition and not return to play without the clearance of a health care provider (*10*). Continuing to play with a concussion might worsen symptoms (*1*) and increase the risk for a second concussion; therefore, it is crucial to talk with athletes about the importance of reporting their concussion symptoms (*10*). Among recreational activities, bicycle helmets have been shown to reduce head injuries (*1*). There is a need to expand programs, policies, and practices, tailored to specific audiences, to ensure that all students, parents, coaches, teachers, and health care providers know how to prevent, recognize, and manage concussions. It is critical that these stakeholders know how to safely return students to school and to play following a concussion.

**Summary**

**What is already known about this topic?**

A concussion is a type of traumatic brain injury. Surveillance efforts likely miss some concussions related to sports and physical activity among youths.

**What is added by this report?**

The 2017 Youth Risk Behavior Survey found that 15.1% of students reported having at least one concussion related to sports or physical activity, and 6.0% reported having two or more. Playing on more than one sports team was found to further increase the risk for concussion.

**What are the implications for public health practice?**

It is important to expand education about the risk for concussion, the signs and symptoms of concussion, the need to remove athletes with a suspected concussion from play, the evaluation of concussions, and the protocol for athletes’ safe return to school and play.

## Footnotes

* The National Center for Education Statistics estimated that in 2017, a total of 16,451,000 youths were enrolled in public and private high schools. This number was multiplied by the estimated prevalence of one or more and then two or more concussions related to sports or physical activity found in this study.

† Overall response rate = school response rate x student response rate.

§ https://nces.ed.gov/pubs2016/2016117rev.pdf.
